# Do plasmid-dependent phages enable the survival of costly plasmids?

**DOI:** 10.64898/2026.06.01.729315

**Published:** 2026-06-02

**Authors:** Jonathon L. Yuly, Matthew Avallone, Lawrence Abad, Ned S. Wingreen

**Affiliations:** 1 Lewis-Sigler Institute for Integrative Genomics, Princeton University, Princeton, NJ 08540; 2 Department of Molecular Biology, Princeton University, Princeton NJ 08540

## Abstract

Plasmids benefit bacterial communities by storing auxiliary genes that address environmental challenges such as antibiotics. Subsequent plasmid loss can also be advantageous if plasmid benefits are temporary but costs are permanent. However, unless positive selection is sustained, plasmid loss can proceed to extinction, with access to plasmid-derived benefits permanently lost. In principle, horizontal transmission can maintain a plasmid in a population, but if the plasmid cost is too high, the host can become uncompetitive. We examine how survival of costly but occasionally beneficial plasmids is possible in a bacterial population. Using population models, we demonstrate that plasmid-dependent phages can, counterintuitively, solve this plasmid survival problem for their bacterial hosts. Phage predation pins the plasmid at low but nonzero abundance, such that the plasmid cost is effectively neutralized at the population level, dramatically lengthening the persistence time of the plasmid. When conditions change and the costly plasmid becomes beneficial, it spreads across the host population and switches to a vertical-transmission lifestyle until benefits again subside.

Bacteria in nature face fierce competition across diverse and often rapidly changing environments. To help them survive, bacteria harbor and exchange plasmids, small genetic elements (typically ~ 10 – 100 kb [1]) that can encode auxiliary genes to address transient or niche environmental challenges [2, 3, 4, 5]. For example, if a community is challenged by an antibiotic or toxin, plasmids with genes that provide resistance can spread and rescue the population. Similarly, if a niche energy or nutrient source is encountered, plasmids can spread that encode alternate metabolic pathways [6, 7, 8]. Should these environmental challenges subside, the population can lose the plasmid and any associated costs without relying on chromosomal modifications. However, positive selection for a plasmid auxiliary gene might be very occasional, such as the appearance of an antibiotic that might not be encountered again for a long time. How do bacteria prevent complete plasmid loss during long periods between selection events, even many thousands of generations?

Several mechanisms are known to promote long-term plasmid persistence, including fitness enhancing interactions between plasmids [9, 10], fitness enhancing compensatory evolution of host chromosome and plasmid genes [11, 12, 13, 14], sufficiently frequent positive selection events [15], “weaponization” of the plasmid against competitors [16], and sustained beneficial plasmid effects for a fraction of the host population [17]. However, all of these mechanisms succeed by decreasing the cost of the plasmid or by providing sufficiently sustained positive selection. Is long-term persistence possible for plasmids with genes that provide only infrequent benefits and impose high costs that cannot be alleviated?

To survive, plasmids have evolved mechanisms that prevent loss from hosts, especially during cell division. For example, plasmids can tune their copy number in the cell [18], actively partition plasmids between daughter cells [19], and deploy addiction systems to kill cells that do not retain the plasmid [20]. Thus, the probability of plasmid loss after cell division is reported as low as 10^−7^ [21]. Plasmids also spread horizontally from donor bacteria to acceptor bacteria via conjugation [22]. This exchange of genetic material is facilitated by some Type IV secretion systems (T4SS) [23]. However, the ubiquity of these extracellular structures makes them attractive receptors for phages, specifically plasmid-dependent or “male specific” phages that target conjugation machinery, especially the conjugative pilus [24]. Early studies suggested that conjugation rates were too slow to sustain costly plasmids even against small loss rates [25, 26, 27], but evidence is accumulating of sufficiently high conjugation rates even in lab cultures [28, 29, 30, 31]. Are the mechanisms for plasmid spread and persistence sufficient to enable the long-term survival of plasmids that carry high-cost genes? How does phage predation impact these dynamics?

Population modeling of plasmids and their hosts is routinely used to understand cell growth and plasmid abundance in experiments [32, 33], establish criteria for plasmid fixation in a population [34, 26, 35], and more [36, 37]. Here we use this modeling framework to clarify the fate of high-cost plasmids in the presence of plasmid-dependent phage. We find that (1) horizontal transfer and persistence mechanisms are alone unlikely to enable the long-term survival of high-cost plasmids, but that (2) plasmid-dependent phages can promote the long-term survival of high-cost plasmids. Our results are robust within realistic parameter ranges and yield several testable predictions.

## Results

### Long-term survival of costly plasmids is unexpected

High-cost plasmid genes face seemingly insurmountable odds against long-term survival. We first discuss this survival qualitatively and then quantitatively evaluate the timescale for high-cost plasmid loss within a population model. Finally, we demonstrate how plasmid-dependent phages can rescue high-cost plasmids from rapid extinction.

**Figure 1:**
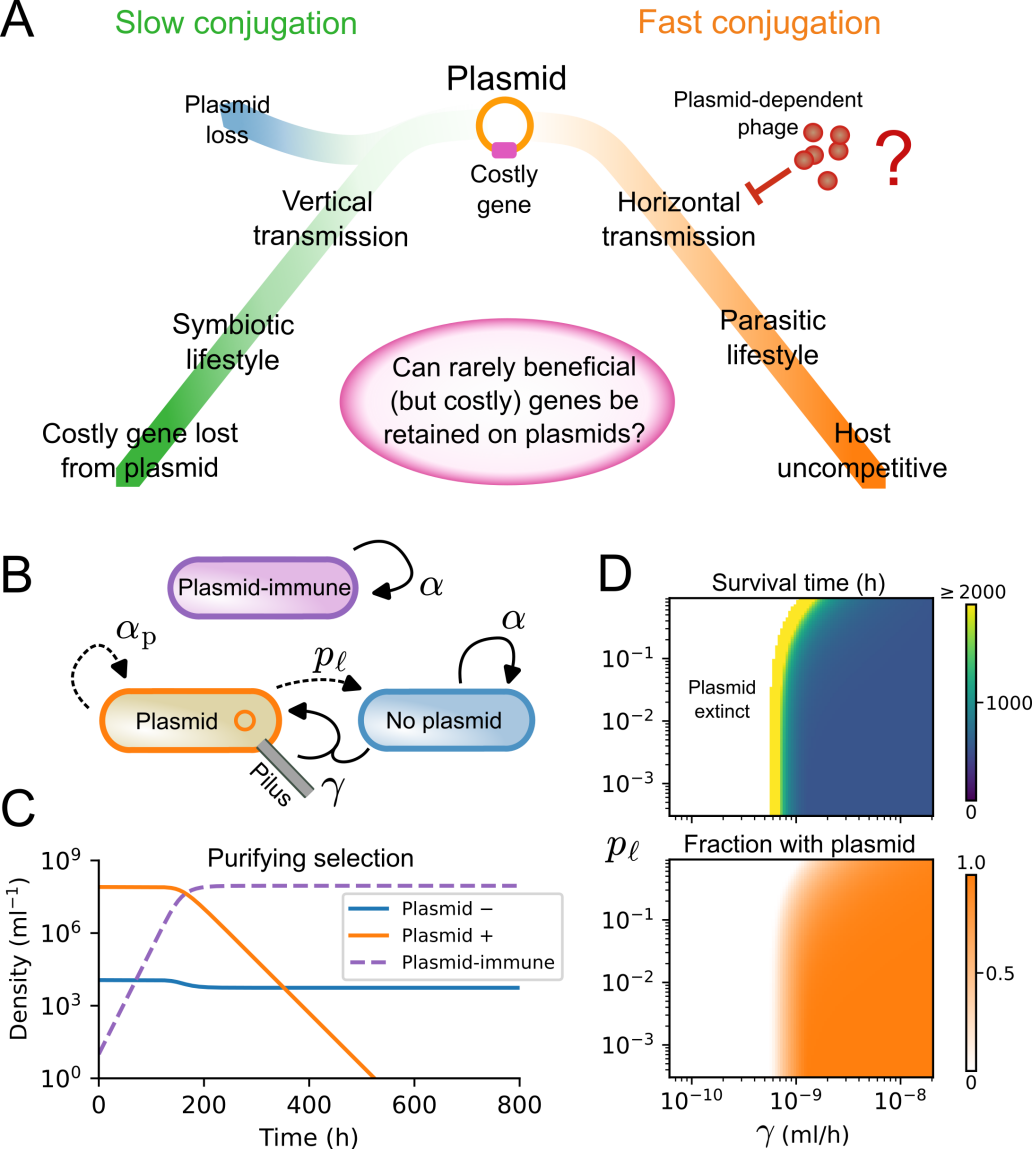
Can bacterial communities retain costly genes on plasmids until the genes become useful? (A) When horizontal transfer via conjugation is slow, plasmid survival depends on vertical transmission (symbiosis) and costly genes are purified out of the plasmid. If conjugation is frequent instead, plasmid takeover can render the host uncompetitive. However, plasmid-dependent phages may disrupt this pathway. (B) A model ecology with a single costly plasmid that spreads quickly via conjugation (rate $\gamma_{hi}=10^{-8}ml\;{hr}^{-1}$) but cannot infect a subpopulation of cells (plasmid-immune) and is lost upon cell division with probability $p_{\ell }=10^{-3}$. Plasmid-containing cells grow at a rate $\alpha_{p}=0.5/h$ that is 50% slower than plasmid-free cells α=1.0/h. All growth is logistic with a carrying capacity $Q=10^{8}/ml$ and a death/washout rate of δ=0.1/hr. (C) At equilibrium (t=0) plasmid-containing cells (orange) dominate the susceptible population (blue = plasmid free). The plasmid-immune competitor (dashed purple) is introduced and drives the plasmid to extinction, which occurs when abundance drops below 1.0/ml. (D) The survival time (top) of the plasmid following invasion by the competitor is always small, unless the conjugation and plasmid-loss rates are extremely finely balanced to produce an initial steady state (bottom) with only a small fraction of plasmid containing cells.

High-cost genes carried on plasmids are in constant danger of extinction. If horizontal transmission of a costly plasmid is too slow to overcome loss rates (Figure 1A, left), plasmid extinction is guaranteed unless the plasmid harbors other genes that provide a benefit greater than the cost. In this symbiotic regime, selection will drive the loss of costly genes, while beneficial genes are expected to migrate to the host chromosome [27]. Rapid horizontal transfer is also insufficient to save the costly gene(s). In the high-conjugation regime (Figure 1A, right), the plasmid will quickly spread to fixation as a parasite within the susceptible population. However, this rapid spread ultimately undermines plasmid survival because the plasmid cost leaves the host uncompetitive against plasmid-immune populations. Such plasmid-immune competitors could include bacteria with entry exclusion mechanisms [38], restriction enzymes targeting the costly plasmid [39, 40], or even archaeal or eukaryotic competitors.

To estimate the extinction timescales for costly plasmids, we employ a population model (illustrated in Figure 1B) of a plasmid-susceptible population under invasion by a plasmid-immune competitor. Plasmid-free and plasmid-immune cells both grow logistically at a maximum rate of α=1.0/hr, describing an ecology that is competitive in the absence of the plasmid. The maximum growth rate for plasmid-containing cells is $\alpha_{p}=(1-\Delta )\alpha$ where Δ=0.5 is the plasmid growth cost. This 50% growth penalty describes a very costly plasmid. The plasmid can spread horizontally (conjugation rate γ) and is lost upon cell division with probability $p_{\ell }=10^{-3}$. See Methods and SI Appendix for details.

When the conjugation rate is high enough, the plasmid can initially fix in the susceptible population. Starting with an invasion by a plasmid-immune competitor (t=0, Figure 1C), within ~ 500 h, the plasmid population goes extinct (extinction threshold 1/ml), allowing the susceptible population to continue but without any long-term benefits the plasmid may carry. The plasmid-immune competitor population is the critical factor driving costly plasmid loss in the high-conjugation regime of Figure 1C. In models without plasmid-immune competitors [41, 35] and presumably experiments on lab cultures [30], long-term persistence can be sustained by high conjugation rates alone. However, we expect plasmid-immune competitors to be ubiquitous in natural environments.

Figure 1D plots the time to extinction as a function of γ and $p_{\ell }$. The survival time of the plasmid is very short, except for a narrow region where the conjugation and loss probabilities are extremely finely balanced such that plasmid-containing cells constitute only a small fraction of the susceptible population at steady state (t=0). In Figure 1D, the timescale for extinction increases as the the plasmid-containing fraction decreases because the impact of the plasmid scales with the plasmid-containing fraction. More precisely, the plasmid-susceptible cells (both plasmid-containing and plasmid-free) grow subject to the effective growth cost (SI Appendix 1.2)

(1) $$\Delta_{eff}=p\Delta ,$$

where p is the plasmid-containing fraction and ∆ is the growth penalty of the plasmid. In the next section, we show that a plasmid-dependent phage can promote the survival of costly plasmids by keeping the plasmid-containing fraction low (and therefore reducing the impact of the plasmid on the population).

### Predation-enabled plasmid persistence

**Figure 2:**
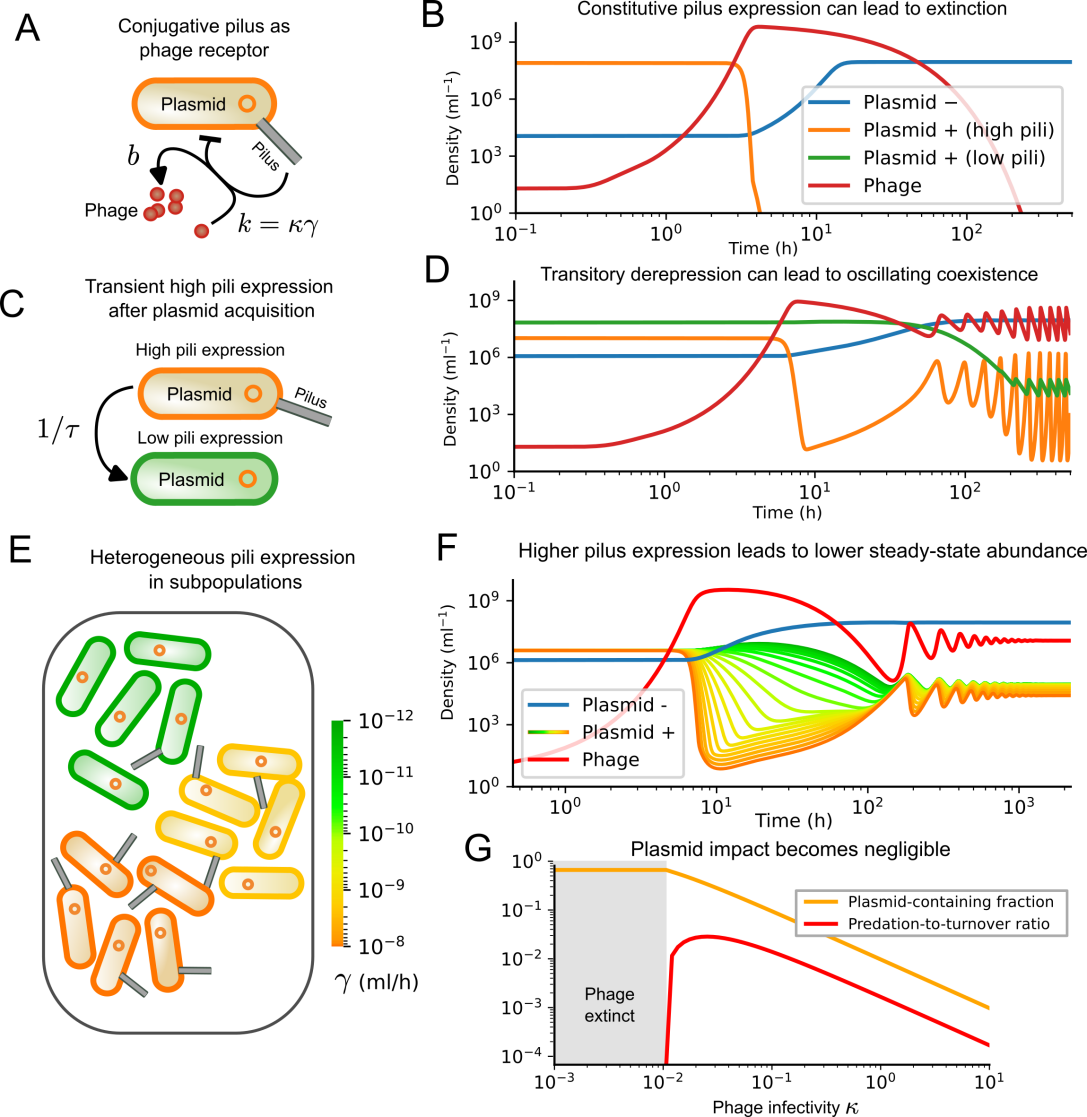
When plasmid-dependent phages are present, costly plasmids are maintained at low abundance. (A) Plasmid-dependent phages target conjugative pili as surface receptors. We assume that the phage absorption rate k and the conjugation rate γ are proportional (k=κγ). (B) When conjugative pili are constitutively expressed (conjugation rate $\gamma_{hi}=10^{-8}ml/hr,\kappa =1$), phage invasion can result in plasmid extinction (occurring when populations drop below 1/ml). To avoid extinction, a simple pilus regulatory strategy is to (C) only transiently express pili at a maximum level ($\gamma_{hi}$) for an average time τ after plasmid acquisition, then dramatically reduce expression ($\gamma_{lo}=10^{-12}ml/hr$). This regulation (D) prevents plasmid extinction but can lead to oscillations. Heterogeneity often dampens such oscillatory dynamics in nature, and we describe such heterogeneity by dividing the population into N=20 subpopulations with constitutive expression of conjugation machinery to cover the range $\gamma_{hi}\leq \gamma \leq \gamma_{lo}$. (F) Heterogeneity dampens the oscillations, with high-expression cell populations driven to lower abundance than low-expression populations. (G) As the phage infectivity (κ) increases above the phage survival threshold ($\kappa \approx 10^{-2}$), the plasmid-containing fraction (orange) falls by orders-of-magnitude. Counterintuitively, losses due to phage predation also decrease (red, showing the ratio of phage infection to death/washout rate without the phage).

Plasmid-dependent phages target conjugative machinery (especially conjugative pili) as surface receptors, and therefore may achieve a broad host range [42], up to the range of plasmids they target. We focus specifically on lytic plasmid-dependent phages, many of which have recently been discovered [43, 44]. By introducing a plasmid-dependent phage into our population model (illustrated in Figure 2A) we demonstrate how predation by these phages can dramatically extend survival of high-cost plasmids. We refer to this dynamic as “predation-enabled plasmid persistence”.

To describe the phage population in our model, we use dynamics that are routinely used in other contexts to model phage populations (see SI Appendix). Since the expression of conjugation machinery determines both the conjugation and phage infection kinetics, we assume that the phage infection rate k and the conjugation rate γ are proportional [45] (k=κγ, where κ is the proportionality constant). We use parameter values that are typical for conjugation and lytic phage infection kinetics (see Methods).

We first examine the dynamics of several invasions of plasmid-dependent phages into a bacterial population with a single costly plasmid, without plasmid-immune competitors (Figure 2A). In the invasion of Figure 2B, expression of conjugation machinery is constitutive such that conjugation is always occurring at the maximum rate (see Methods). The model and conditions are the same as in Figure 1C, but with the addition of the phage. In this case, the plasmid is driven to extinction by the phage. However, such extinction can be avoided when expression of the conjugation machinery is regulated [22] (not constitutive) or heterogeneous within the population [45].

First, we consider phage invasion when conjugation is regulated by a minimal framework called transitory de-repression [28], illustrated in Figure 2C. In this mechanism, transconjugants express conjugation machinery at high levels (achieving a fast conjugation rate $\gamma_{hi}$) for only an average time τ before repressing the machinery permanently to low levels (conjugation rate $\gamma_{\text{lo}}$). Thus, conjugation is primarily driven by recent transconjugants. Even this simple regulatory framework can prevent plasmid extinction by phage predation, as shown in Figure 2D. Extinction is prevented and plasmid abundance is maintained at a low level, even as $\gamma_{lo}$ and τ vary over orders-of-magnitude (see Figure S1). In the limit-cycle dynamics of Figure 2D, the population of plasmid-containing cells oscillates with frequency $\omega \approx \sqrt{\delta_{P}\left(\gamma_{hi}Q-1/\tau \right)}$ around a fixed point with population $\approx \delta /b\kappa \gamma_{hi}$, where δ is the death (washout) rate of the phage and b is the lysis burst size (see SI Appendix 2.1).

Next, we consider a population with heterogeneous expression of conjugation machinery, as illustrated in Figure 2E. There are many reasons that bacterial populations (even monoclonal) may express conjugation machinery heterogeneously. For example, expression of conjugation machinery has been observed to vary depending on cell activity [46], plasmid “interference” [47], quorum sensing [48], and more [22]. To describe heterogeneity in pilus expression, we evenly bin (on a log scale) the initial plasmid-susceptible population by average conjugation rate γ into N=20 groups that vary from low to high pili expression (conjugation rate). Within each group, cells either have the plasmid or not. As a result of this heterogeneity, the oscillations in plasmid abundance are dampened and a steady state is reached after a few hundred hours. Subpopulations with faster conjugation (higher pilus expression) are suppressed to lower abundance. Prior modeling studies describing conjugation heterogeneity and plasmid-dependent phages [45] are consistent with the results in Figure 2, but did not consider how these phages might promote plasmid survival.

The impact of plasmid-dependent phages on costly plasmids in the above examples suggests a generalization with potentially far-reaching consequences: after initial transient dynamics, plasmid-dependent phages pin down the abundance of plasmid-containing cells to low levels, but not to extinction. This drives down the effective growth cost $\Delta_{\text{eff}}$ of the plasmid to the susceptible population (Equation 1). However, this raises another question – to what extent is the effective fitness boost to the bacteria due to smaller growth costs counteracted by an effective fitness decrease driven by cell losses via phage predation?

To evaluate the impact of predation losses on the plasmid-susceptible population, we evaluate the steady-state behavior of Figure 2F as a function of the phage infectivity κ. Figure 2G shows both the plasmid-containing fraction (orange) and the ratio ρ (red)

(2) $$\rho =\frac{R_{P}}{Q\delta },$$

where $R_{P}$ is the total rate of phage infections across the susceptible population, and Qδ is the death (washout) rate of the population when it fills the carrying capacity Q. Therefore, ρ captures the relative magnitudes of cell loss due to death (washout) versus phage predation. At low infectivity, the phage population cannot be sustained and goes extinct, allowing the plasmid to spread to fixation in the population. As the infectivity κ increases to a value typical for lytic phages (κ∼1 or higher), the plasmid-containing fraction falls dramatically to well below a percent. Counterintuitively, the rate of phage predation ρ also decreases by orders of magnitude because predation limits prey abundance. For typical infection rates of lytic phages ($k_{hi}=10^{-7}ml/h$ or κ=10), the phage negligibly increases the total turnover rate of the plasmid-susceptible population.

**Figure 3:**
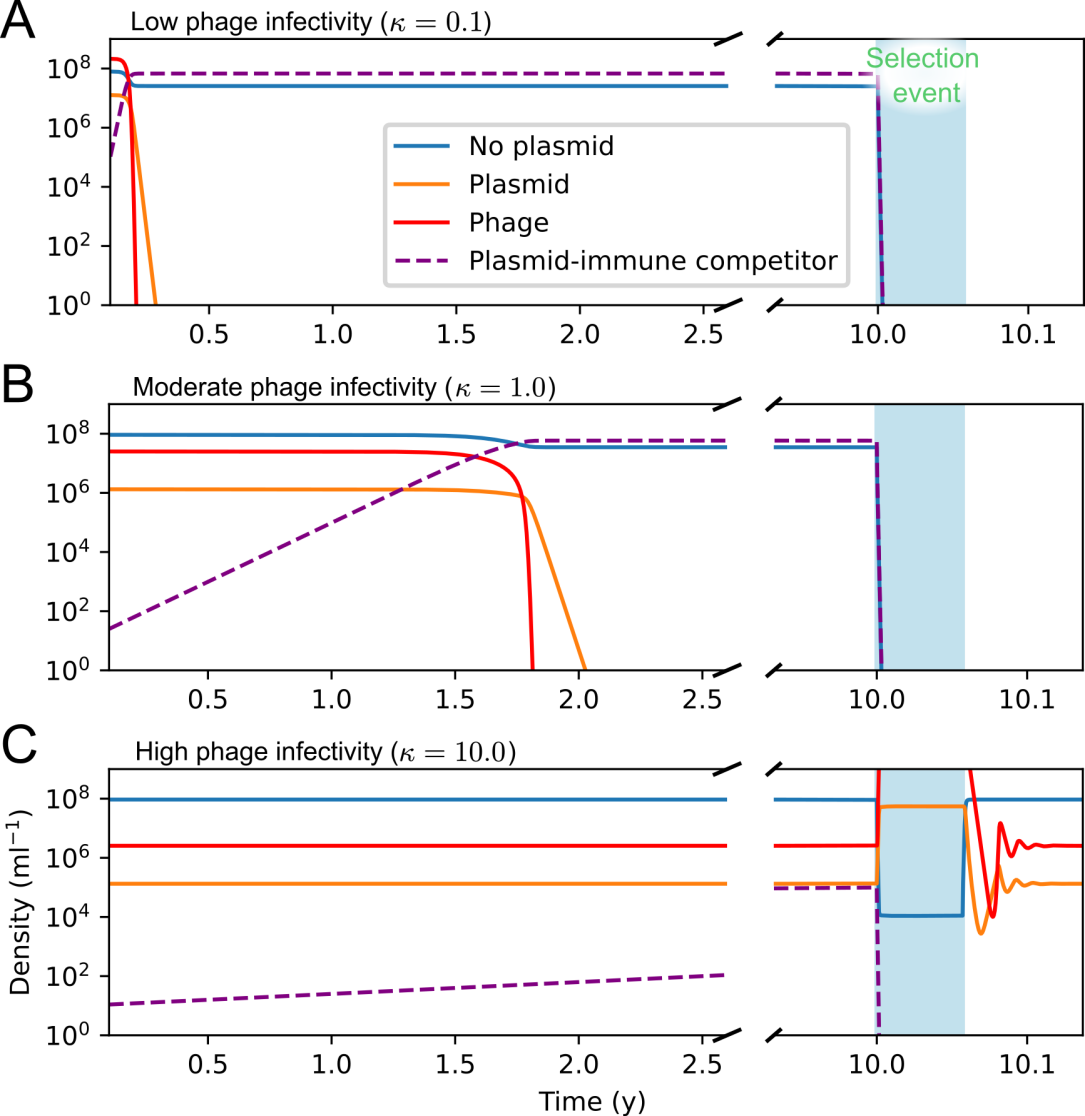
Plasmid-dependent phages can enable costly plasmids to survive between rare selection events. In this example, survival of the bacteria requires the persistence of a costly antibiotic resistance plasmid until the appearance of an antibiotic at t=10 years (selection event, shown in light blue). The antibiotic kills plasmid-free cells at a rate 2.0/h. (A) Even at low infectivity κ=0.1 ($k_{\text{hi}\;}=\kappa \gamma_{\text{hi}\;}=10^{-9}ml/h$) the plasmid persists for ~ 2000 h, much longer than in the absence of the phage (Figure 1C), but far too short to reach the selection event, so all cells as well as the plasmid and phage die. As phage infectivity increases from (B) κ=1.0 $\left(k_{\text{max}}=10^{-8}ml/h\right)$ to (C) κ=10.0 $\left(k_{\text{max}\;}=10^{-7}ml/h\right)$, the costly plasmid imposes progressively less and less of a burden on the host population, slowing the timescale of plasmid extinction by years. In (C), the plasmid survives long enough to provide protection from the antibiotic. The model and other parameter values are the same as in Figures 1C, 2F, and 2G.

The results in Figure 2 demonstrate that plasmid-dependent phages can in principle confine high-cost plasmids to low abundance – but not extinction – without any fine tuning or special regulation, and simultaneously inflict minimal predation losses as a result. This regime is precisely where persistence of the costly plasmid is maximized. Figure 3 illustrates how phage predation can enable costly plasmid survival for many thousands of generations until conditions recur in which the plasmid can yield its benefits.

In the example of Figure 3, the plasmid-susceptible population must hold onto a high-cost plasmid for 10 years, after which a plasmid selection event occurs (i.e. the appearance of an antibiotic). During this selection event, cells without the plasmid are killed at a rate $r_{s}=2.0/h$. If the plasmid survives until the event, it can rescue the susceptible population and ultimately allow the susceptible population to outcompete the plasmid-immune competitor that cannot receive protection from the plasmid. As phage infectivity is increased from low (κ=0.1) to high (κ=10.0, still within typical ranges, see Methods), the timescale for invasion of the plasmid immune competitor and subsequent plasmid extinction is increased from ~ 1000 h to many years (κ=10.0). When the selection event occurs, the costly plasmid quickly switches to a vertical-transmission lifestyle, allowing low-conjugation subpopulations to dominate.

After the selection event, the survival of the plasmid depends on further ecological details. In particular, high-conjugation cells must be replaced after each selection event for the plasmid to survive across many selection epochs. Otherwise, the phage predation during the selection event can drive extinction of the high-conjugation populations during the selection event (see Figure S2). Several mechanisms for replacement of high-conjugation cells are possible, such as mutations in the regulation of conjugative machinery. In figure 2E–G, we show the case of slow interconversion of conjugation states ($T_{\text{mix}}=100\;h$), reflecting changes in expression of conjugation machinery over time driven by external factors. Importantly, the specifics of positive selection events do not impact the central conclusion of this study: plasmid-dependent phages can enable the survival of costly plasmids for long periods between selection events.

## Discussion

Plasmids can be critical for the survival of bacterial communities in nature by yielding genetic flexibility in the face of unpredictable environmental changes [2, 3, 4]. Plasmids can be harnessed when needed, and disposed of when not needed. For this strategy to work, however, plasmids must not be lost entirely between beneficial periods. Survival of high-cost plasmids is especially difficult, as purifying selection is expected to purge them from populations (Figure 1). We propose that plasmid-dependent phages can, counterintuitively, promote the survival of their target plasmids by suppressing plasmids to low, but nonzero, abundance within their host populations. We observed this behavior in a population model with typical (and varied) parameter values (Figures 2–3).

The emergence of predation-dependent persistence of high-cost plasmids relies on two key features in our analysis: (1) the presence of plasmid-immune competitors, and (2) the negative feedback inherent in predator-prey dynamics. With plasmid-immune competitors, costly plasmids can only survive for long periods when the conjugation and loss kinetics are unrealistically finely balanced to maintain the plasmid at low abundance (Figure 1D). While previous analyses considered plasmid-dependent phages to only decrease the likelihood of plasmid persistence [45, 49], we reach a different conclusion: by predating on cells expressing conjugative pili, plasmid-dependent phages can establish a critical negative feedback: as plasmid abundance increases, so does phage predation, and when plasmid abundance falls, phage predation also falls. This ecological feedback can suppress high-cost plasmid populations to low but nonzero levels (with no need for fine tuning), precisely the conditions required for plasmids to survive for long periods (Figure 3).

Beyond the lytic plasmid-dependent phages we analyzed, other sources of ecological feedback could lead to suppression of high-cost plasmids and thus render them almost neutral at the population level. For example, some plasmid-dependent phages are filamentous and spread by secretion of phage particles from infected hosts, instead of lysing their hosts to spread [50]. Diverting cellular resources to produce phage particles (or occlusion of the conjugative pilus by phage [51]) may limit the conjugation rates of host bacteria, leading to falling abundance of plasmid-containing cells. As infected cells become more rare, so do the filamentous phages, and conjugation rates rise again. This behavior may constitute another negative feedback loop that can constrain the spread of high-cost plasmids without the need for fine tuning.

Our theory yields at least three testable predictions: (1) High-cost plasmids (~ 50% growth costs or even more) can exist in nature where plasmid-dependent phages are found. These plasmids must be sustained by tiny bacterial subpopulations with high conjugation rates. Such “super donor” populations have been previously considered [29, 45, 41], but previous analyses did not consider the possibility of predation-enabled plasmid persistence. (2) Introducing plasmid-dependent phages to cultures where they are absent can drive an increase in average bacterial fitness (such as growth rates), by cutting down the abundance of any costly plasmids. (3) Plasmid-dependent phages can be used as a capture reagent for rare and costly plasmids, which we discuss next.

Our findings support a picture of the mobilome where high-risk, high-reward genes are maintained in a bacterial community at low abundance, a potential hidden reservoir of genes that conventional metagenomics approaches fail to capture. In our models, plasmid-dependent phages tend to target high-cost plasmids, because these plasmids are forced to express conjugation machinery at high levels to survive. Thus, plasmid-dependent phages themselves might be used for high-cost plasmid discovery. For example, lysis-deficient plasmid-dependent phages could be used to immobilize otherwise flowing cells that express conjugation machinery. This would create a sample of bacteria greatly enriched for plasmids in this low-abundance reservoir. Already, similar experiments have demonstrated trapping of bacteria expressing T4 receptors [52, 53]. Alternatively, plasmid-dependent phage vectors [54] could be used to deliver antibiotic resistance markers to cells expressing conjugation machinery, allowing these plasmid-containing cells to be selected out of a population.

More broadly, we anticipate that significant revisions may be forthcoming to our understanding of plasmid-dependent phages and their role in natural bacterial communities. Our results strongly motivate further experimental and theoretical investigation, especially focusing on small bacterial subpopulations. Finally, our results recall a familiar ecological motif: the impacts of predation on an ecosystem can be both unexpected and dramatic [55, 56, 57].

## Methods

Our models combine routine approaches to plasmid conjugation kinetics [34, 34, 35, 36] with Lotka-Volterra style predation dynamics describing the plasmid-dependent phage [45], where we include 0.5 h delays between infection and lysis and between plasmid acquisition and pilus expression (see SI Appendix for details). We solve these equations using an automatic stiffness detection method [58] implemented in the SciPy [59] wrapper of ODEPACK [60].

Unless otherwise stated, we use $\alpha =1.0/h,\alpha_{p}=0.5/h,Q=10^{8}/ml,p_{\ell }=10^{-3}$, and κ=1. In all simulations, we set the extinction threshold to 1.0/ml. To estimate the value of the phage infectivity parameter κ, we assume that the phage infection kinetics correspond to those typical for lytic phages. Specifically, pilus expression for the F plasmid can be permanently derepressed to yield a maximum conjugation rate of around $\gamma_{hi}\approx 2\times 10^{-9}ml/hr$ [61] to 3×10^−8^ ml/h [32]. Thus, we use $\gamma_{hi}∼10^{-8}\;ml/hr$, although conjugation rates as high as 10^−6^ ml/hr have been reported for some plasmids [62]. We expect that phages absorb to these fully derepressed cells at rates typical for lytic phages. Phage absorption rates are typically $k∼10^{-7}ml/h$ or less [63]. Thus, the “infectivity constant” κ in our model should be $\kappa =\gamma /k∼10^{1}$ or less. Unless stated otherwise, we conservatively use κ=1. A value $\kappa \approx 10^{-4}$ was previously suggested [45], but such infection rates would be anomalously low among lytic phages, was derived assuming homogeneity in experimental bacterial populations, and predicts phage extinction where it was not observed [64]. During the selection events of Figure 3, all cells without the plasmid die at a rate 2.0/h.

## Supplementary Material

Supplement 1
